# Respiratory Muscle Training and Its Impact on Balance and Gait in Patients with Severe COPD

**DOI:** 10.3390/medicina60020257

**Published:** 2024-02-01

**Authors:** Crisan Alexandru Florian, Pescaru Camelia Corina, Maritescu Adelina, Carunta Vlad, Oancea Cristian, Vastag Emanuela

**Affiliations:** 1 Research Center for the Assessment of Human Motion, Functionality and Disability (CEMFD), “Victor Babes” University of Medicine and Pharmacy Timisoara, Eftimie Murgu Square 2, 300041 Timisoara, Romania; crisan@umft.ro; 2 Pulmonary Rehabilitation Center, Clinical Hospital of Infectious Diseases and Pulmonology, “Victor Babes”, Gheorghe Adam Street 13, 300310 Timisoara, Romania; adelina.maritescu@umft.ro; 3 Center for Research and Innovation in Personalized Medicine of Respiratory Diseases (CRIPMRD), “Victor Babes” University of Medicine and Pharmacy Timisoara, Eftimie Murgu Square 2, 300041 Timisoara, Romania; oancea@umft.ro (O.C.); emanuela.tudorache@umft.ro (V.E.); 4 Doctoral School, “Victor Babes” University of Medicine and Pharmacy Timisoara, Eftimie Murgu Square 2, 300041 Timisoara, Romania; 5 Faculty of Physical Education and Sports, West University of Timisoara, Vasile Parvan Street 4, 300223 Timisoara, Romania; carunta.vlad@gmail.com; 6 Pulmonology Clinic, Clinical Hospital of Infectious Diseases and Pulmonology, “Victor Babes”, Gheorghe Adam Street 13, 300310 Timisoara, Romania

**Keywords:** COPD, respiratory muscle training, balance, activities confidence, gait

## Abstract

*Background and Objectives*: Improving extrapulmonary symptoms in COPD through respiratory muscle training can help alleviate the burden of respiratory symptoms, reduce fatigue, and improve exercise capacity in patients with COPD. This, in turn, can enhance physical activity, balance, and gait, ultimately improving the overall quality of life for individuals with COPD. This study aimed to investigate the effects of respiratory muscle training on balance and gait in patients with moderate to severe COPD. *Materials and Methods*: We included 65 patients with moderate to severe COPD randomly assigned to either the pulmonary rehabilitation protocol group (PR) or the pulmonary rehabilitation and inspiratory muscle training group (PR + IMT) for three weeks. Patients performed a spirometry, maximal inspiratory and expiratory pressure (MIP/MEP), 6 min walking test (6MWT), activities-specific balance confidence (ABC) scale questionnaire, Berg Balance Scale (BBS), timed up and go test (TUG), and single-leg stance test (SLS). *Results*: Rehabilitation had a notable impact on MIP in Group 2 (PR + IMT), with a highly significant difference between pre- and post-rehabilitation distributions (*p* < 0.0001). At the same time, Group 1 (PR-only) showed no significant changes (*p* = 0.27). In Group 1 (Control), pre- and post-rehabilitation comparisons reveal slight non-significant changes for SLS EO (*p* = 0.16), ABC (*p* = 0.07), TUG (*p* = 0.06), and BBS (*p* = 0.13). In contrast, in Group 2 (Cases), there are significant improvements in all variables after rehabilitation compared to the pre-rehabilitation values: SLS EO (*p* < 0.0001), ABC (*p* < 0.0001), TUG (*p* < 0.0001), and BBS (*p* < 0.0001). *Conclusions*: Our research demonstrated that respiratory muscle training significantly positively impacts balance and gait performance among patients with moderate to severe COPD compared to a control group.

## 1. Introduction

Patients with chronic obstructive pulmonary disease (COPD) often experience dyspnea, a reduction in exercise capacity, and a decline in their overall quality of life. These symptoms are prevalent and have a significant impact on patient’s well-being [1].

The most widely recognized and studied mechanisms contributing to respiratory muscle dysfunction are chest wall geometry and diaphragm position alterations.

COPD is a diverse disease with numerous extrapulmonary effects, often resulting in frequent impairment of balance function among patients. This impairment is closely linked to functional challenges in daily activities and increased susceptibility to falls [2].

To maintain a high quality of life and mitigate the risk of falls, it is crucial to prioritize the preservation of balance function in individuals with COPD. Consequently, the guidelines set forth by the American Thoracic Society and European Respiratory Society strongly recommend assessing balance function following pulmonary rehabilitation in COPD patients [2,3].

Studies have demonstrated that, when faced with heightened respiratory demands, the diaphragm prioritizes its role in breathing over its role in posture [3,4].

Patients with COPD face intrinsic risk factors that increase their susceptibility to falls, including weakness in the muscles of the lower limbs and deficits in gait and balance [1,5].

We consider that inspiratory muscle training (IMT) will significantly improve balance and gait performance in patients with moderate to severe COPD compared to a control group, suggesting that targeted interventions can positively impact extrapulmonary manifestations of the disease. Thus, this study aims to investigate the effects of respiratory muscle training on balance and gait in patients with moderate to severe COPD.

## 2. Materials and Methods

This study enrolled chronic COPD patients seeking treatment in our Pulmonary Rehabilitation Centre. The criteria for patient inclusion were history of smoking of more than 20 packs per year, forced expiratory volume (FEV1) less than 80% of predicted, FEV1/forced vital capacity (FVC) ratio less than 0.7 predicted, and a physician diagnosis of COPD [6].

The patients were excluded from the study if they had severe comorbidities or acute conditions that could potentially interfere with their existing health status, medication that could potentially affect their exercise responses, as well as those diagnosed with postural orthostatic hypotension, neurological or musculoskeletal conditions that may contribute to falls and balance issues (such as Parkinson’s disease, a history of cerebrovascular accident, acute or severe cardiovascular problems, transient ischemic attacks, or recent lower-extremity joint replacements), patients with exacerbations in the last 3 months or that were oxygen dependent, and individuals with impaired vision. Our study included 65 patients with moderate to severe COPD.

Patients who met the inclusion criteria were randomly assigned to either the pulmonary rehabilitation protocol group (PR) or the pulmonary rehabilitation and inspiratory muscle training group (PR + IMT). Both groups underwent a comprehensive rehabilitation protocol twice a day for three weeks.

### 2.1. Lung Volumes and Respiratory Muscle Strength

We conducted assessments of lung volumes, as well as maximal inspiratory and expiratory pressure (MIP/MEP). To assess pulmonary volumes and measure maximal inspiratory and expiratory pressure, we utilized the Smart PFT UI device provided by Medical Equipment Europe GmbH, Hammelburg, Germany.

For assessing maximal expiratory pressure, the patient was guided to inhale until reaching total lung capacity, after which they were instructed to exhale forcefully. Three measurements were taken, and the highest value was recorded. All maneuvers were executed following standard procedures [7].

### 2.2. Balance Assessment

Before the balance tests, patients were asked to fill out the activities-specific balance confidence (ABC) scale questionnaire, which consists of 16 items assessing various daily living tasks, including basic activities and more challenging ones performed in the community [8,9].

After completing the questionnaire, the patients underwent the Berg Balance Scale (BBS) assessment. The BBS is a widely used 14-item scale that evaluates performance in simple balance tasks. The test duration is approximately 15 min, with each task being assessed using a 5-point ordinal scale ranging from 0 to 4. A score of 0 signifies an inability to perform the task, while a score of 4 indicates independent completion [10].

Afterward, the participants underwent the timed up and go test (TUG), a performance measure that evaluates general mobility and includes balance and gait maneuvers relevant to everyday activities [11]. Previously, a validated cutoff value of 8.42 s in the timed up and go (TUG) test indicated average physical capacity in patients with COPD [12].

The single-leg stance test (SLS) was performed to assess static balance. This test measures the duration a participant could maintain a stance on one leg without assistance. In individuals aged 60–75 years, SLS test scores below 30 s, regardless of whether the eyes are opened or closed, are deemed abnormal [13].

To evaluate the functional exercise level, we used the 6 min walking test (6MWT) following the guidelines set by the American Thoracic Society (ATS) [14].

### 2.3. Pulmonary Rehabilitation Program

The pulmonary rehabilitation program included thoracic expansion exercises, coughing techniques, diaphragmatic breathing exercises, oscillatory positive expiratory pressure (OPEP) devices, and counseling on physical activity. The exercises were conducted in two sets, consisting of 5–7 repetitions each, with rest intervals of 5–6 tidal breaths between practices to prevent respiratory muscle fatigue and hyperventilation, which is under the ERS/ATS guidelines [15].

The endurance exercise training program involved three weeks with one daily session for both groups. This particular segment of the training program focused on supervised aerobic exercises. Each session consisted of 30 min of treadmill exercise. The session concluded with stretching exercises for both the upper and lower limbs. Each participant received an individualized program tailored to their abilities, targeting 50% to 70% of the average speed achieved during the six-minute walk test [16].

The strength exercise training involved daily sessions for both groups with 1–3 sets of 8–12 repetitions with an initial load equivalent to 60–80% of the one maximum repetition (1RM), as recommended by the American College of Sports Medicine [17].

### 2.4. Inspiratory Muscle Training

The program included two daily sessions consisting of 30 breaths for the rehabilitation program period. The intensity of the training load was gradually increased every week, reaching the highest tolerable intensity, starting from 40% of MIP values and reaching up to 60% in the last week of the program.

### 2.5. Statistical Analysis

The statistical analysis was performed using the Python programming language and data analysis libraries, including Pandas (v2.1.3), NumPy (v1.26.0), and Scipy (v1.11.3). Continuous variables were reported as mean + standard deviation or median (interquartile range), depending on the normality of the distribution. Categorical variables are reported as the absolute counts (percentage). Comparison of continuous variables was performed with a *t*-test or Mann–Whitney U test; for categorical variables, comparison was performed using Fisher’s exact test. Kolmogorov–Smirnov tests were used to compare the distributions of the variables of interest. Univariate and multivariate binomial logistic regressions were used to determine the likelihood of having an elevated level of activities-specific balance confidence by group. *p* < 0.05 was considered statistically significant.

## 3. Results

### 3.1. Baseline Characteristics

The mean age of the 65 COPD patients included in the study was 64.73 years. The participants’ mean FVC (L) was 2.83, and the mean FEV1 (L) was 1.16. The results showed no significant differences in age, height, weight, BMI, FVC, FEV1, FVC percentage, and FEV1 percentage between the two groups. Notably, a significant difference was observed in the FEV1/FVC ratio (*p* = 0.01) (Table 1).

### 3.2. Respiratory Muscle Strength

Overall, the median MIP increased from 58 cmH_2_O in the pre-rehabilitation phase to 66.6 cmH_2_O in the post-rehabilitation phase. Similarly, the mean MEP increased from 77.10 cmH_2_O (68% of predicted) in the pre-rehabilitation phase to 83 cmH_2_O in the post-rehabilitation phase.

For Group 1 (Control- PR only), the median MIP was 60.24 cmH_2_O (58% predicted) in the pre-rehabilitation phase and increased to 65.60 cmH_2_O (64% predicted) in the post-rehabilitation phase. The mean MEP was 77.65 cmH_2_O (68% of predicted) in the pre-rehabilitation phase and increased to 81.80 cmH_2_O (72.50% of predicted) in the post-rehabilitation phase. While the changes showed a trend towards improvement, they were not statistically significant, with *p*-values ranging from 0.07 to 0.15. In contrast, for Group 2 (Cases), significant improvements were observed in MIP and MEP after rehabilitation. The *p*-values for comparing pre- and post-rehabilitation values within Group 2 were highly significant (*p* < 0.0001 to *p* = 0.0005).

**Figure 1:**
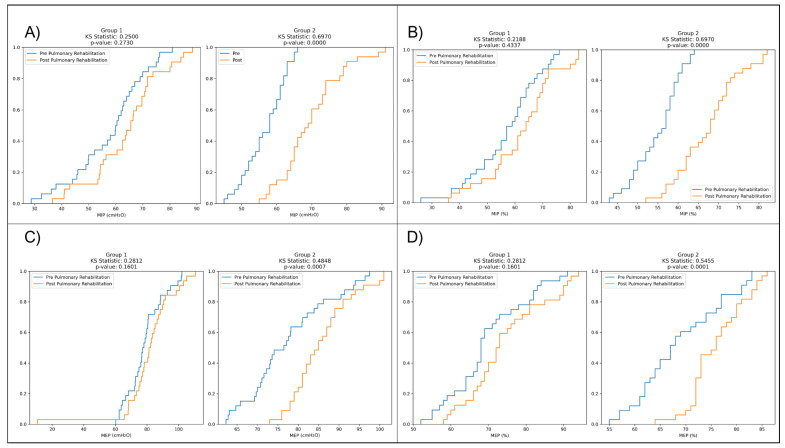
Kolmogorov–Smirnov (KS) plots comparing pre- and post-rehabilitation distributions of: (**A**) MIP (cmH_2_O) in two groups; (**B**) MIP (%) in two groups; (**C**) MEP (cmH_2_O) in two groups; (**D**) MEP (%) in two groups.

Furthermore, the Kolmogorov–Smirnov (KS) plots demonstrated that rehabilitation had a notable impact on MIP in Group 2 (PR + IMT), with a highly significant difference between pre- and post-rehabilitation distributions (*p* < 0.0001), while Group 1 (PR-only) showed no significant changes (*p* = 0.27). Similar findings were observed for MEP distributions, with Group 2 (PR + IMT) displaying highly significant differences in pre- and post-rehabilitation distributions (*p* = 0.0007), unlike Group 1 (*p* = 0.16) (Figure 1).

### 3.3. Balance Assessment

Overall, in the entire sample, the median distance covered during the 6-minute walk test (6MWT) increased from 340 m (75% predicted) in the pre-rehabilitation phase to 375 m (83% predicted) in the post-rehabilitation phase.

For Group 1 (Control), the median distance covered during the pre-rehabilitation 6MWT was 354.5 m, increasing to 377.5 m in the post-rehabilitation phase. However, these changes were not statistically significant.

For Group 2 (Cases-PR + IMT), the median distance covered during the pre-rehabilitation 6MWT was 325 m, and it significantly increased to 375 m in the post-rehabilitation phase. The *p*-values for the comparison between pre- and post-rehabilitation within Group 2 were 0.0007 and 0.0001 for the distance covered (in meters) and the percentage of predicted, respectively, indicating significant improvements in the 6MWT performance after rehabilitation.

**Figure 2:**
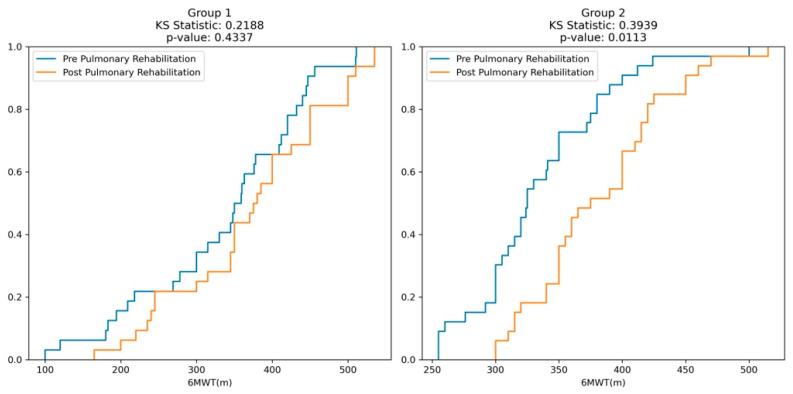
Kolmogorov–Smirnov (KS) plots comparing pre- and post-rehabilitation distributions of 6WT(m) in two groups.

Moreover, in Group 1 (Control), for 6MWT in meters, the KS statistic is 0.21 (*p* = 0.43), indicating a non-significant difference between the pre- and post-rehabilitation distributions. Similarly, for 6MWT as a percentage of predicted values, the KS statistic is 0.12 (*p* = 0.98), reinforcing the lack of significant differences. In contrast, for Group 2 (Cases), the KS plots for both 6MWT in meters and percentage show higher KS statistics of 0.39 (*p* = 0.01) and 0.51 (*p* = 0.0002), respectively, signifying statistically significant differences between the pre- and post-rehabilitation distributions (Figure 2).

Table 2 presents a comparison of pre- and post-rehabilitation outcomes for single-leg stance with eyes open (SLS EO) in seconds, activities-specific balance confidence (ABC) in percentage, timed up and go (TUG) in seconds, and Berg Balance Scale (BBS) in points.

However, in Group 1 (Control), pre- and post-rehabilitation comparisons reveal slight non-significant changes for SLS EO (*p* = 0.16), ABC (*p* = 0.07), TUG (*p* = 0.06), and BBS (*p* = 0.13).

In contrast, in Group 2 (Cases), there are significant improvements in all variables after rehabilitation compared to the pre-rehabilitation values: SLS EO (*p* < 0.0001), ABC (*p* < 0.0001), TUG (*p* < 0.0001), and BBS (*p* < 0.0001) (Table 2).

**Figure 3:**
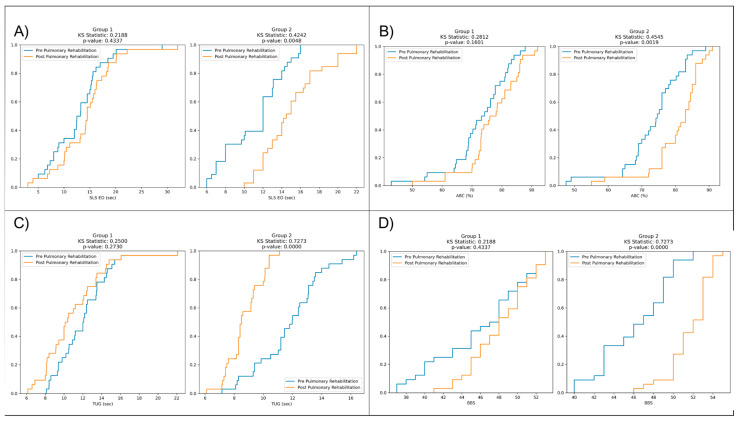
Comparison of pre- and post-rehabilitation distributions of (**A**) single-leg stance with eyes open (SLS EO) in Group 1 and Group 2; (**B**) activities-specific balance confidence (ABC) in Group 1 and Group 2; (**C**) timed up and go (TUG) in Group 1 and Group 2; (**D**) Berg Balance Scale (BBS) distributions in Group 1 and Group 2.

Furthermore, in Group 1, the KS statistic for SLS EO (sec) is 0.21 with a *p*-value of 0.43, indicating no significant difference between the pre- and post-rehabilitation distributions. Conversely, in Group 2, the KS statistic is 0.42, with a highly significant *p*-value of 0.004, suggesting substantial differences between the pre- and post-rehabilitation distributions (Figure 3).

Furthermore, in Group 1, the KS statistic for ABC (%) is 0.28, with a *p*-value of 0.16, indicating no significant difference between the distribution before and after rehabilitation. However, in Group 2, the KS statistic is 0.45, accompanied by a highly significant *p*-value of 0.001, suggesting considerable differences between pre- and post-rehabilitation distributions.

In Group 1, the KS statistic for TUG (sec) is 0.25, with a *p*-value of 0.27, suggesting no significant difference between the pre- and post-rehabilitation distributions. This result implies that the rehabilitation intervention had a similar effect on timed up and go performance for Group 1 participants, with no notable changes in the TUG times between the pre- and post-rehabilitation assessments.

Moreover, in Group 1, the KS statistic for BBS is 0.21, with a *p*-value of 0.43, indicating no significant difference between the distribution before and after rehabilitation. This result suggests that the rehabilitation intervention had a similar effect on BBS scores for participants in Group 1, with no notable changes in balance scale scores between pre- and post-rehabilitation assessments.

In contrast, in Group 2, the KS statistic for BBS is 0.72, accompanied by a highly significant *p*-value of 0.000. This finding demonstrates substantial differences between the distribution of pre- and post-rehabilitation BBS scores in Group 2. The rehabilitation program had a remarkable effect on improving balance and stability, resulting in significantly higher BBS scores for participants in Group 2 after the intervention (Figure 3).

## 4. Discussion

Our research aimed to establish that IMT effectively enhances balance and gait performance in patients with moderate to severe COPD compared to a control group.

The results of our study indicate a noteworthy enhancement in functional balance as measured by the BBS and ABC scores in the intervention group, suggesting that the addition of IMT to PR may contribute to improved balance.

Including IMT in PR resulted in notable improvements in BBS scores by 3.6 points, along with a corresponding increase of 3.7 points in ABC scores between the groups by the end of the program. We also observed significant correlations between inspiratory muscle strength and BBS and ABC scores. However, these correlations were only significant in the intervention group. These findings align with a previous study by Beauchamp et al. [2] who reported a significant 2.8-point improvement in BBS scores following six weeks of pulmonary rehabilitation in a similar group. Furthermore, the experimental group, which underwent both PR and balance training, demonstrated superior results to the control group, which solely underwent PR.

These findings can also be attributed to the increase in MEP values observed in patients who underwent IMT, with the intervention group experiencing an improvement of 8.1 cmH_2_O compared to 2.5 cmH_2_O in the control group. This aligns with Zeren et al. [4] who discovered that the MEP value independently predicts dynamic postural stability. It is also worth noting that the MEP value reflects the combined impact of the abdominal and internal intercostal muscles and the elastic recoil of the lungs and chest wall [4].

Furthermore, we detected significant differences in the TUG and SLS tests, indicating a reduction in completion time for the TUG tests and an improvement in performance for the SLS tests. Specifically, the TUG tests exhibited a 2.2 s difference between the groups, favoring the PR + IMT group. Additionally, the SLS test demonstrated a 2.4 s increase in performance time within the same group.

The findings from the TUG test align with previous studies by Beauchamp et al. [2] and Marques et al. [18]. Beauchamp et al. reported a small but significant difference in TUG scores (15.7 s to 14.2 s) within the same group before and after six weeks of pulmonary rehabilitation in individuals with COPD. Similarly, Marques et al. observed a difference in TUG scores from 8.9 s to 7.2 s in the experimental group.

Regarding inspiratory muscle training, the findings of this research revealed a substantial improvement in inspiratory muscle strength (MIP) among patients who underwent IMT as an additional intervention. Based on the research conducted by Foskolou et al. [3], which highlights the impact of simple diaphragmatic breathing instructions, physical therapists are also guided to promote active diaphragmatic engagement during IMT. The study revealed that incorporating such instructions resulted in a noteworthy augmentation of diaphragm recruitment during IMT, in contrast to IMT sessions without specific guidance [3].

In our study, we observed a notable increase of 13 cmH_2_O in MIP after three weeks of PR + IMT, with intensity progressively set from 40% to 60% of MIP, compared to 6.3 cmH_2_O in the PR-only group. The observed improvement in inspiratory muscle strength can be attributed to the adaptive structural modifications within the inspiratory muscles. According to Develi et al. [19], an increase in inspiratory muscle strength plays a crucial role in maintaining the equilibrium between intra-pleural and intra-abdominal pressures, in line with the physiological ventilation mechanism. Consequently, higher MIP values are likely to contribute to improved dynamic balance.

Although both groups demonstrated noticeable enhancements in the 6MWT, with the PR + IMT group showing an increase of 47 m and the PR-only group showing an increase of 36 m, the incorporation of IMT into the PR program resulted in minimal to negligible improvements in the 6MWT. The average mean difference between the groups was only 9 m. Our findings are similar to the research conducted by Charususin et al. [20], where the authors demonstrated a lack of significant difference in the 6MWT between the experimental group (IMT + PR) and the control group (PR only) within the context of a pulmonary rehabilitation program combined with IMT.

The results of our study demonstrate a significant improvement in various measurements for the intervention group compared to the control group. These include a noteworthy increase in BBS and ABC scores (3.6 and 3.7 points), improvements in TUG and SLS test performance (2.2 and 2.4 s), enhanced MIP and MEP values (13 cmH_2_O and 8.1 cmH_2_O), as well as a substantial increase of 47 m in the 6MWT distance. Consequently, these findings support the hypothesis that IMT positively affects balance and gait performance compared to the control group.

While our results reveal notable improvements in functional balance and stability among moderate-to-severe COPD patients, the increase in balance can be attributed to several potential physiological mechanisms that play a vital role.

One possible mechanism involves the stimulation of the diaphragm and intercostal muscles, prompting them to adapt and respond to diverse movements and frequencies. This adaptation is believed to aid in maintaining balance during swift and destabilizing motions of the upper body. Foskolou et al. [3] proved that the diaphragm regulates postural stability during abrupt voluntary limb movements. However, it is worth noting that the postural activity of the diaphragm may decrease with an increase in respiratory demand. In addition to the diaphragm, the intercostal muscles have been demonstrated to participate in postural control, extending their role beyond breathing [21]. These muscles contribute to maintaining balance, specifically during rotational movements of the thorax [22].

Guo et al. [22] demonstrated that the diaphragm and abdominal muscles coordinate to maintain increased intra-abdominal pressure during postural and trunk movements. Consequently, it is plausible that the diaphragm plays a role in trunk postural control by elevating the intra-abdominal pressure.

Our research findings align with these potential physiological mechanisms and support the idea that enhancing inspiratory muscle strength improves functional balance. Furthermore, this improvement in balance may aid in the recovery of compromised balance caused by increased trunk muscle activity.

The three-week intervention period may have limitations in capturing the long-term effects of inspiratory muscle training on gait and balance in COPD patients. A longer follow-up duration would provide a more comprehensive understanding of the sustained benefits and potential relapses without continued training.

## 5. Conclusions

Our research demonstrated that respiratory muscle training significantly positively impacts balance and gait performance among patients with moderate to severe COPD compared to a control group. This suggests incorporating inspiratory muscle training into pulmonary rehabilitation may also enhance balance.

## Figures and Tables

**Table 1:** Baseline characteristics of included patients.

Parameters	Overall (*n* = 65)	Group 1—Control (*n* = 32)	Group 2—Cases (*n* = 33)	*p*-Value
Age	64.73 + 4.77	64.43 + 4.48	65.03 + 5.08	0.78
Height (cm)	171.06 + 6.78	170.84 + 7.26	171.27 + 6.38	0.81
Weight (kg)	74.26 + 17.92	73.68 + 19.81	74.81 + 16.17	0.65
BMI	24.9 (6.5)	23.95 (11.85)	25.10 (5.30)	0.77
FVC (L)	2.83 + 0.50	2.81 + 0.51	2.85 + 0.51	0.59
FVC (%)	70.13 + 7.51	69.56 + 6.72	70.69 + 8.26	0.63
FEV1 (L)	1.16 + 1.16	1.22 + 0.24	1.10 + 0.28	0.06
FEV (%)	34 (13)	34.00 (12.25)	35.00 (14.00)	0.25
FEV1/FVC (%)	41.15 + 7.90	43.57 + 5.74	38.80 + 9.02	0.01

**Table 2:** Comparison of balance results between control group (Group 1) and cases group (Group 2) pre- and post-rehabilitation.

		SLS EO (sec)	ABC (%)	TUG (sec)	BBS (Points)
Overall	Pre-rehabilitation	12 (6.5)	74.20 (10.60)	12.01 (3.25)	47 (6)
	Post-rehabilitation	14.5 (5)	80.50 (11.7)	9.20 (2.2)	50 (5)
Group 1 Control (*n* = 32)	Pre-rehabilitation	12.85 (6.75)	73.7 (11.2)	12.05 (3.4)	47.5 (7.5)
	Post-rehabilitation	14.5 (6.21)	77.05 (10.65)	10.26 (4.37)	48 (4.5)
	*p*-value	0.16	0.07	0.06	0.13
Group 2 Cases (*n* = 33)	Pre-rehabilitation	12 (5.10)	74.6 (9.5)	11.98 (2.6)	47 (6)
	Post-rehabilitation	14.5 (4.5)	83 (10)	8.50 (1.25)	52 (3)
	*p*-value	0.0001	0.0001	<0.0001	<0.0001

## Data Availability

The supporting data for the findings of this study can be obtained by contacting the corresponding author upon request.
